# Phosphorylation of Mutant Huntingtin at Serine 116 Modulates Neuronal Toxicity

**DOI:** 10.1371/journal.pone.0088284

**Published:** 2014-02-05

**Authors:** Erin E. Watkin, Nicolas Arbez, Elaine Waldron-Roby, Robert O'Meally, Tamara Ratovitski, Robert N. Cole, Christopher A. Ross

**Affiliations:** 1 Division of Neurobiology, Department of Psychiatry, Johns Hopkins University School of Medicine, Baltimore, Maryland, United States of America; 2 Mass Spectrometry and Proteomics Facility, Johns Hopkins University School of Medicine, Baltimore, Maryland, United States of America; 3 Departments of Neurology, Pharmacology and Neuroscience and Program in Cellular and Molecular Medicine, Johns Hopkins University School of Medicine, Baltimore, Maryland, United States of America; University of Florida, United States of America

## Abstract

Phosphorylation has been shown to have a significant impact on expanded huntingtin-mediated cellular toxicity. Several phosphorylation sites have been identified on the huntingtin (Htt) protein. To find new potential therapeutic targets for Huntington's Disease (HD), we used mass spectrometry to identify novel phosphorylation sites on N-terminal Htt, expressed in HEK293 cells. Using site-directed mutagenesis we introduced alterations of phosphorylation sites in a N586 Htt construct containing 82 polyglutamine repeats. The effects of these alterations on expanded Htt toxicity were evaluated in primary neurons using a nuclear condensation assay and a direct time-lapse imaging of neuronal death. As a result of these studies, we identified several novel phosphorylation sites, validated several known sites, and discovered one phospho-null alteration, S116A, that had a protective effect against expanded polyglutamine-mediated cellular toxicity. The results suggest that S116 is a potential therapeutic target, and indicate that our screening method is useful for identifying candidate phosphorylation sites.

## Introduction

Huntington's disease (HD) is a fatal progressive neurodegenerative disorder involving movement, cognitive and emotional symptoms, with no current neuroprotective therapy [1]–[10]. The striatum is the main structure of the brain affected by the neurodegeneration, but some is also notable in the cortex and other brain regions, especially in early onset cases or late stage disease [11]–[14]. HD is caused by a CAG triplet repeat expansion in the *Huntingtin* gene on chromosome 4 coding for a polyglutamine repeat expansion in the Huntingtin protein (Htt) [15]. There is a correlation between repeat length and the severity and age of onset of the disease. Longer repeats cause earlier onset and more widespread neurodegeneration.

The pathogenesis of HD is still incompletely understood, but is believed to arise predominantly via a genetic gain of toxic function due to the CAG repeat expansion [9], [16], [17]. The polyglutamine (polyQ) expansion in the Htt protein results in change in its conformation and metabolism. The expanded protein can be cleaved into N-terminal fragments, which in most experimental systems, are more toxic that full-length Htt [18]–[22]. A cleavage by caspase 6 at position 586 is believed to be one of the first steps of the toxic proteolysis of Htt [23]. Transgenic mouse models expressing the caspase 6 fragment or other shorter fragments generally have more striking and robust phenotypes than transgenic mouse models expressing full-length Htt [20], [24]–[27]. Downstream steps in the pathogenic process likely include nuclear localization and accumulation resulting in alterations of transcription, abnormal proteostasis, and interference with metabolic and mitochondrial function. These disruptions leave the cell compromised and sensitive to stress (e.g. oxidative stress) [1]. The conformational changes and aggregation of mutant Htt caused by the polyQ expansion has been extensively observed in human post-mortem brain and mouse models. These aggregates are characteristically present as nuclear inclusions [24], [28], as well as aggregates elsewhere in the cell. The relationship between aggregation and cell toxicity is complex (e.g. [28]–[30]). Neuronal cell death in HD has some features of apoptosis with nuclear condensation and fragmentation, neurite retraction and caspase activity [11], [31], [32]. A recent model of inducible pluripotent cells derived from human HD patients also recapitulated many of those features [33].

Htt is a very large protein with many protein interactions, and likely with many normal functions in the cell [9], [16], [34]–[38]. There are many sites of post-translational modification, including phosphorylation, which can have substantial effects on mutant Htt cell biology, cellular localization, cleavage and cell toxicity [1], [39]–[45]. Phosphorylation of serine 421 by Akt or SGK [46] regulates the involvement of Htt in axonal transport [47], [48]. Phosphorylation of serine 421 also reduces the nuclear accumulation and cleavage of huntingtin [49], and protects against neuronal toxicity [50]–[53]. Phosphorylation at positions 434, 1181 and 1201 by Cdk5 has also been reported to be protective [54], [55].

The N-terminal 17 amino acids of Htt, being immediately adjacent to the polyglutamine repeat, appear to be especially important for Htt pathogenesis [56]. Phosphorylation of residues in the N-terminal 17 amino acids (threonine 3 and serines 13 and 16) can alter Htt conformation and reduce toxicity *in vitro* and *in vivo* [57]–[65]. Beside those few identified phosphorylation sites many other potential sites on Htt remain unknown.

As the region of the protein near the N-terminal polyglutamine repeat may be especially important for pathogenesis, we sought to determine additional sites of phosphorylation of Htt within this region. We used immunoprecipitation and mass spectrometry to identify novel phosphorylation sites within an N-terminal region fragment ending shortly before the known caspase cleavage sites. We found a number of candidate sites, and screened them for modulation of toxicity. In addition to the confirmation of known phosphorylation sites, we find that that alteration of the serine at position 116 has striking modulatory effects on Htt cell toxicity.

## Materials and Methods

### Antibodies and reagents

Goat polyclonal 909 antibody, prepared against the N-terminal Htt exon-1 fragment was described previously [66]; Htt monoclonal MAB5492 antibody (against residues 1–82 of Htt) was from Millipore. Actin antibody was from Sigma. Ro 31–8220 was purchased from Sigma.

### Animals

For primary neurons, CD1 mice were purchased from Jackson Laboratory. This study was carried out in strict accordance with the recommendations in the Guide for the Care and Use of Laboratory Animals of the National Institutes of Health. The protocol was approved by the Johns Hopkins Animal Care and Use Committee (Protocol MO09M411).

### Cell culture and transfection

Human embryonic kidney (HEK) 293FT cells were from Invitrogen. Cells were kept in DMEM (with 4.5 g/L D-Glucose, Invitrogen) supplemented with 10% FBS, 100 units/ml penicillin/streptomycin. (GIBCO), and 100 µg/ml Geneticin in 5% CO_2_ at 37°C. Cells were split at a 1/10 ratio when reaching 95% confluency. For Western blot experiments, cells were plated in 6 well plates (Corning) and for mass spectrometry experiment cells were plated in 100 mm dishes (Corning). Primary mouse cortical neurons were prepared as described previously [32]. Cortices of CD1 mice at embryonic day 15.5 were dissected out, treated with trypsin (0.05% with EDTA, GIBCO) and mechanically dissociated. Neurons were suspended in Neurobasal medium supplemented with B27 (Invitrogen) and plated at 1×10^6^ cell/cm^2^ on poly-D-lysine coated 24 well plates (Corning). Cells were kept in Neurobasal medium supplemented with B27 and 2 mM GlutaMAX (GIBCO) in 5% CO_2_ at 37°C until the day of the experiment. All cells were transfected using Lipofectamine 2000 (Invitrogen) according to the manufacturer's protocol.

### Plasmids and mutagenesis

All Htt constructs used represent N-terminal fragments. They are referred to as N followed by number of amino acids present (e.g. N586). Htt expression constructs N511-52Q, N586-82Q, and N586-22Q were previously described [22]. To generate the N586 phosphorylation mutants, we used the QuikChange Site-Directed Mutagenesis Kit (Agilent) according to the manufacturer's instructions starting with 100 ng of Htt-N586-82Q DNA template and 125 ng of each primer. The sequences of primers used to generate the mutations can be found in Table S1. Primers were synthesized on an Applied Biosystem synthetizer at the Johns Hopkins University School of Medicine Synthesis and Sequencing Facility.

### Purification of Htt Fragments for Mass Spectrometry

HEK 293FT cells were transfected with htt N511-52Q construct. 24 h after transfection, cells were lysed in M-PER buffer (Pierce) with protease inhibitors (Sigma) and with or without phosphatase inhibitors (Pierce, see results). The lysates were diluted 1∶1 with phosphate-buffered saline (PBS) and NaCl was added to a final concentration of 150 mM. FLAG-Htt fusion proteins were immuprecipitated from 20 mg total protein using anti-FLAG M2 affinity gel (Sigma) according to the manufacturer's protocol, followed by elution with 100 µg/mL of FLAG peptide. Eluted proteins were analyzed by Western blot with a FLAG antibody (M2, Sigma). Samples were loaded on NuPAGE 4–12% BisTris polyacrylamide gel, and proteins were visualized with silver stain (SilverQuest kit, Invitrogen).

### In-gel Digestion of Htt Proteins

For mass spectrometric analysis Htt containing bands were manually cut out for in-gel digestion. Gel pieces were destained with 30 mM potassium ferricyanide and 100 mM sodium thiosulfate (50∶50 vol/vol), rinsed with water 3 times, incubated in 20 mM ammonium biocarbonate for 10 min, and dehydrated with acetonitrile. The process was done three times, and gel pieces were then dried in a SpeedVac. For in-gel digestion, gel pieces were incubated overnight at 37°C with 10 ng/l trypsin (Roche) in 20 mM bicarbonate. Peptides were extracted two times with 50% acetonitrile and 2% formic acid. Extracts were pooled and evaporated to dryness.

### LC-MS/MS Analysis

Peptides were analyzed using QSTAR Pulsar (Applied Biosystems-MDS Sciex) interfaced with an Eksigent nano-LC system. Peptides were resuspended in 0.2% formic acid (10 µl). The solution was then separated on a 360×75 µm reverse-phase column of 10 cm of C18 beads (5 µm, 120 Å, YMC ODS-AQ, Waters) and a 10-µm emitter tip (New Objective). The gradient for high pressure liquid chromatography was 5–40% B for 25 min (A, 0.1% formic acid; B, 90% acetonitrile in 0.1% formic acid) at a 300 µL/min flow rate. Survey scans were acquired from m/z 350–1200 with up to three precursors selected for MS/MS using a dynamic exclusion of 30 s. Rolling collision energy was used to promote fragmentation. The electrospray voltage was 900 V and MS/MS spectra of ion 416.7 m/z were acquired for 3 min. The MS/MS spectra were searched against NCLInr database, using Mascot Daemon as an interface and our in-house Mascot server.

### Western blotting

For Western blotting analysis, HEK 293FT cells were lysed 48 h after transfection in M-PER buffer (Pierce) with a protease inhibitors cocktail (Sigma). Protein concentrations were determined using BCA method (Bio-Rad). Proteins were separated on NuPAGE 4–12% BisTris polyacrylamide gels (Invitrogen) and transferred to nitrocellulose membranes (Bio-Rad). After 1 h blocking (PBS, 0.1% Tween 20, 5% fat free dry milk), membranes were probed with primary antibodies, washed, and incubated with peroxidase-conjugated secondary antibodies (Amersham). The signal was detected using chemi-luminescence (ECL-Plus detection reagent, Amersham). Protein bands were quantified using Molecular Imager Gel Doc XR System and Quantity One software (Bio-Rad).

### Cell death assay

To measure the survival of a small population of transfected primary neurons in culture, we established an analysis based on the nuclear condensation observed during cell death. This assay measures the Hoechst staining intensity of transfected neurons. Neurons were co-transfected at DIV5 with the Htt N586-82Q phospho-mutations and eGFP (10∶1 ratio) using Lipofectamine 2000 (Invitrogen) according to the manufacturer's recommendations. After 48 h of expression, cells were fixed with 4% paraformaldehyde for 30 mn and stained with MAB5492 (Htt1-82) antibody according to the laboratory protocol. Nuclei were stained with Hoechst 33258 (bis-benzimide, Sigma-Aldritch). Image acquisition was done using the Axiovision imaging software on an Axiovert 100 inverted microscope (Carl Zeiss) using the automated Mozaix function to cover the integral surface of the wells. Analysis and quantification were performed using the Volocity software (Perkin-Elmer). In order to measure only the intensity of the nuclei of transfected cells, first specific signal of transfected Htt was isolated. Nuclei were subsequently detected in these specific regions corresponding to the transfected cells and their average intensity measured. The percentage of surviving cells was calculated as the percentage of cells for which the average intensity of nuclear staining does not exceed 200% of the average intensity of healthy untransfected nuclei. Results are shown as a percentage of survival of transfected cells, and each independent experiment represent the average of 4 wells per condition.

### Time-lapse imaging

Primary neurons were co-transfected using Lipofectamine 2000 in the same conditions as described above. 38 h post-transfection, plates were transferred into the incubation chamber of an Axiovert 100 inverted microscope (Carl Zeiss) and healthy neurons expressing GFP were randomly selected and followed for 10 h with a picture taken every 10 min. 37°C and 5% CO_2_ were maintained in the chamber throughout the experiment. Morphology of every neuron was quantified blindly using Axiovision software. Cells were given a value for every frame of the experiment: 100 when healthy and 0 when dead. Cells were considered dead when most of the neurites were detached from the soma and fragmented, or when the soma became totally round. Results are expressed as mean ± sem. n = 200 cells analyzed in 5 independent experiments.

### Immunostaining

Cells were fixed directly in the cell culture dishes with PBS containing 4% paraformaldehyde for 30 min. After washing three times with PBS, cells were permeabilized with PBS containing 0.2% Triton X-100 for 10 minutes, then treated with blocking buffer containing 0.2% Triton X-100 and 10% goat serum for 2 h. MAB5492 antibody (1∶1500) was incubated overnight at 4°C in PBS containing 10% goat serum. Cells were rinsed in PBS and incubated for 2 h with anti-mouse CY3-conjugated antibody (1∶200, Jackson). Nuclei were stained with Hoechst 33342 and, after washing twice in PBS, cells were mounted onto slides using Vectashield. Confocal microscopy was performed using a Zeiss Axiovert 200 inverted microscope with 510-Meta confocal module and 63× objective.

## Results

**Figure 1:**
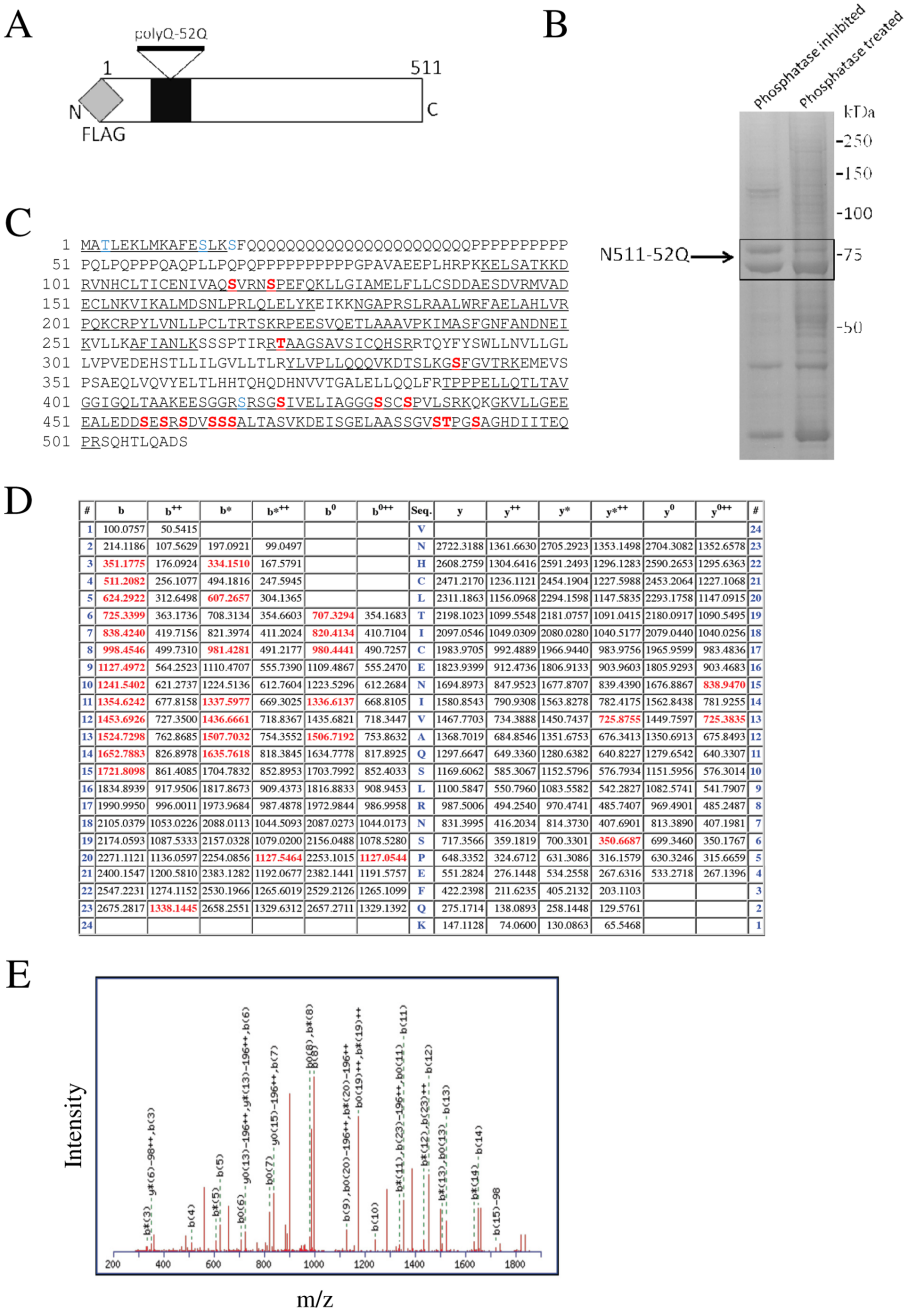
Isolation of Huntingtin N511 for mass spectrometry. (A) Representation of the Htt N511 construct used for transfection. (B) HEK293 cells were transfected with the N511 construct for 24 hours. Protein extracts were immunoprecipitated using Flag antibody and treated with phosphatase inhibitors or phosphatase and separated on a gel. Coomassie Blue detection of proteins in gel shows N511 overexpression. The image shown is representative of 3 replicate experiments. (C) Peptide coverage in two mass spectrometry experiments obtained from trypsin digest of Htt N511. Underlined residues indicate detection, blue indicates previously known phosphorylatable residues, red indicates novel detected sites. (D–E) Examples of mass spectra of peptide showing isotopic distribution for the site at serine 116.

In order to identify novel phosphorylation sites in a region of Htt using mass spectrometry, we chose a fragment of 511 amino acids for study. It is slightly smaller than potential fragments generated by cleavage at the several known caspase sites, and thus is unlikely to be cleaved further by caspases in the cell during the experiment. The construct used contained an N-terminal Flag tag (Figure 1A) and a glutamine repeat length of 52. This is within the expanded range and has toxicity in cells, but does not rapidly cause aggregation in our cell system, which might interfere with immunoprecipitation.

HEK293 cells were transfected with the Htt N511-52Q construct (Figure 1A) and either stimulated with serum and lysed in the presence of phosphatase inhibitors to preserve phosphorylation events, or treated with phosphatase to eliminate any phosphorylation. Htt was purified by immunoprecipitation using antibodies to the Flag tag. The bands containing Htt were identified by Coomassie, and confirmed by Western blot. The phosphatase treatment caused a shift in migration of the expanded Htt protein suggestive of the possibility of alteration by phosphorylation (Figure 1B). Gel slices containing Htt bands were cut out from the gel and processed for mass spectrometry. In some experiments Htt tryptic peptides were enriched for phospho-peptides using a titanium dioxide column. We have obtained around 73% coverage of Htt sequence (Figure 1C) within the region excluding the polyQ and proline repeat region (which does not contain amino acids that would be susceptible to trypsin cleavage). Table 1 shows phosphorylated peptides identified in this experiment. All phosphorylation sites detected were considered for further analysis, even if they had low ion scores, so that we would avoid missing potentially important functional sites. Examples of mass spectra and the fragmentation table of the peptide containing phosphorylated S116 are presented in Figure 1D and 1E.

**Table 1:** Identified phosphorylarion peptides.

Phosphorylated Peptide	Residue	Mascot Score
R.VNHCLTICENIVAQSVRNSPEFQK.L	S116, S120	62
R.TAAGSAVSICQHSR.R	T271	78
R.SRSGSIVELIAGGGSSCSPVLSR.K	S417	70
R.SRSGSIVELIAGGGSSCSPVLSR.K	S421, S431	64
R.SGSIVELIAGGGSSCSPVLSR.K	S421, S434	87
K.VLLGEEEALEDDSESRSDVSSSALTASVK.D	S457	72
K.VLLGEEEALEDDSESRSDVSSSALTASVK.D	S459	92
K.VLLGEEEALEDDSESRSDVSSSALTASVK.D	S461	99
R.SDVSSSALTASVK.D	S464	57
K.VLLGEEEALEDDSESRSDVSSSALTASVK.D	S465, S466	59
K.DEISGELAASSGVSTPGSAGHDIITEQPR.S	S487, T488	70
K.DEISGELAASSGVSTPGSAGHDIITEQPR.S	S491	104

**Figure 2:**
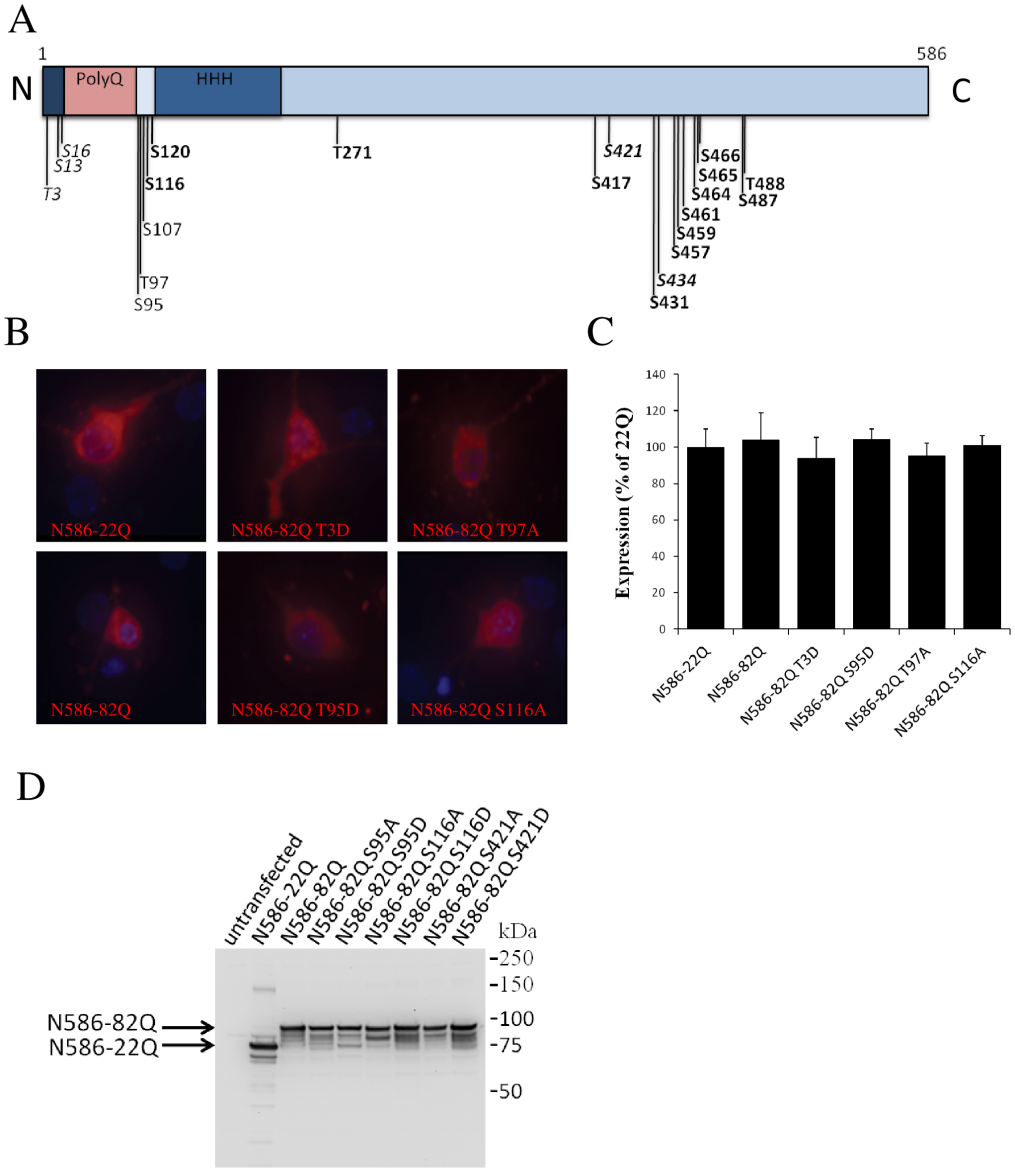
Generation and expression of N586-82Q constructs with phospho-site alterations. (A) Schematic representation of the Htt N586 and the sites altered. Italics indicate previously described phosphorylation sites. Detected sites are in bold. (B) Expression of constructs in primary cortical neurons. (C) Quantification of expression of N586 constructs in primary neurons. Fluorescence was quantified using Volocity, and results are expressed as percent of N586-22Q level of expression. (D) Western blot of expression of N586 constructs with phospho-site alterations in primary cortical neurons. The image shown is representative of 4 replicate experiments.

In order to screen for the potential functional relevance of the detected phosphorylation sites in Htt, we performed site directed mutagenesis to alter all of the sites. Figure 2A shows the sites subjected to site directed mutagenesis. In addition to the sites we identified by mass spectrometry, we also added phosphorylation sites that have been previously described as well as several additional sites in the critical N-terminal region of Htt. For each site, alterations to alanine, which would prevent phosphorylation, and to aspartate, which may mimic phosphorylation, were made. In all cases, at least two clones were generated and tested for expression to confirm that the site directed alterations did not change expression levels.

For our primary functional assay we chose expanded Htt cell toxicity, since this is a highly relevant cell phenotype for this neurodegenerative disease [67]. Our screen for toxicity was done in primary neurons. We chose N586 to do the toxicity experiments since that may be the physiologic fragment *in vivo* [23]. We chose a polyQ repeat length of 82 rather than 52 in order to achieve more robust toxicity, so that we would be able to detect potential reductions of toxicity. Since Htt can cause also cortical cell death and the cortex is affected in HD, we chose to study the toxicity on cortical neurons for their relative abundance. Since it is difficult to do Western blots from transfected cortical neurons, we measured the effect of the site directed alterations in Htt on its expression level by immunofluorescence and quantification of the intensity of fluorescent labeling. Expression level analysis was done at 48 hours, the same time point at which toxicity was measured. The levels of expression of all Htt constructs with alterations in primary cortical neurons were very comparable. Some examples are shown in Figure 2B and 2C. An additional assay to confirm that the constructs did not alter levels of expression was done by transient transfection and Western blot analysis in HEK293 cells (Figure 2D). In our system the expression levels of the N586 fragments with 22Q or 82Q were similar as well.

**Figure 3:**
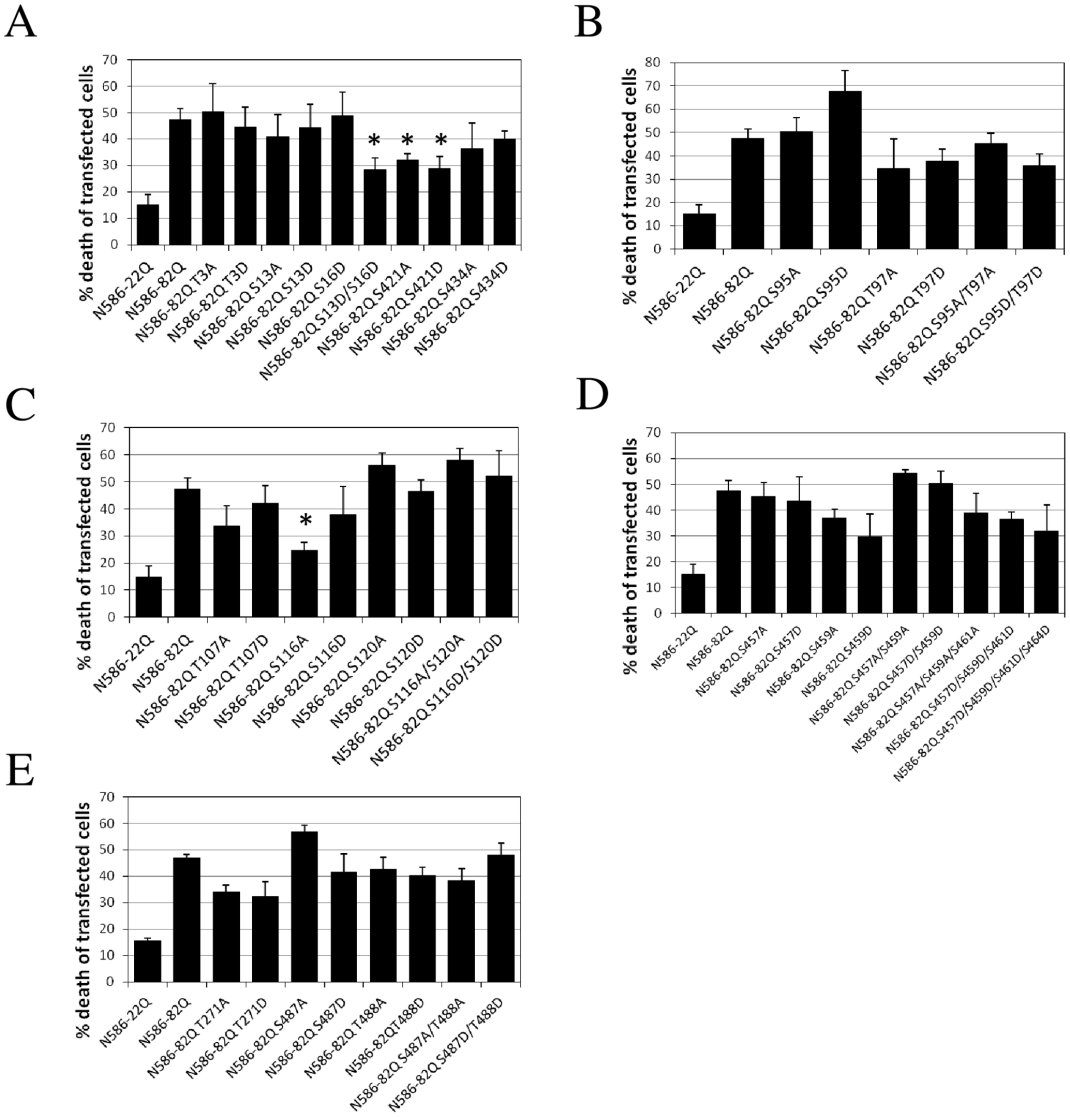
Cell toxicity of N586-82Q constructs with alterations of phospho-sites in primary neurons. Primary cortical neurons were transfected at DIV5. Automated quantification of nuclear condensation was performed 48(A) Toxicity of constructs with alterations of previously identified Htt phosphorylation sites. (B) Toxicity of constructs with alterations of putative Htt phosphorylation sites located between position 1 and 100. (C) Toxicity of constructs with alterations of putative Htt phosphorylation sites located between 100 and 200. (D) Toxicity of constructs with alterations of putative Htt phosphorylation sites located at the 457–464 cluster. (E) Toxicity of constructs with alterations of putative Htt phosphorylation sites located between 200 and 586. Results are expressed as mean ± sem. n = 5 independent experiments per condition. * p<0.05 compared to N586-82Q.

The results of the screen for Htt toxicity are shown in Figure 3. We used a nuclear condensation assay which correlates well with neuronal cell death induced by Htt [32]. We observed robust cell death induced by Htt N586-82Q compared to baseline cell toxicity observed for Htt N586-22Q. As had been previously demonstrated, the change of serine 421 to aspartate caused reduced Htt toxicity, however we also observed a reduced toxicity of Htt with an alteration to an alanine at position 421. Because previous studies had shown that serine 13 and serine 16 tend to be phosphorylated in tandem, we also have included a double mutant of S13D/S16D. This construct showed significantly reduced cell toxicity. None of the other novel candidate sites that we identified showed significant effect on expanded Htt cell toxicity, except for serine 116. Strikingly, change of serine 116 to alanine in Htt results in significantly decreased cell toxicity in the nuclear condensation assay. By contrast, change to aspartate had no significant effect.

**Figure 4:**
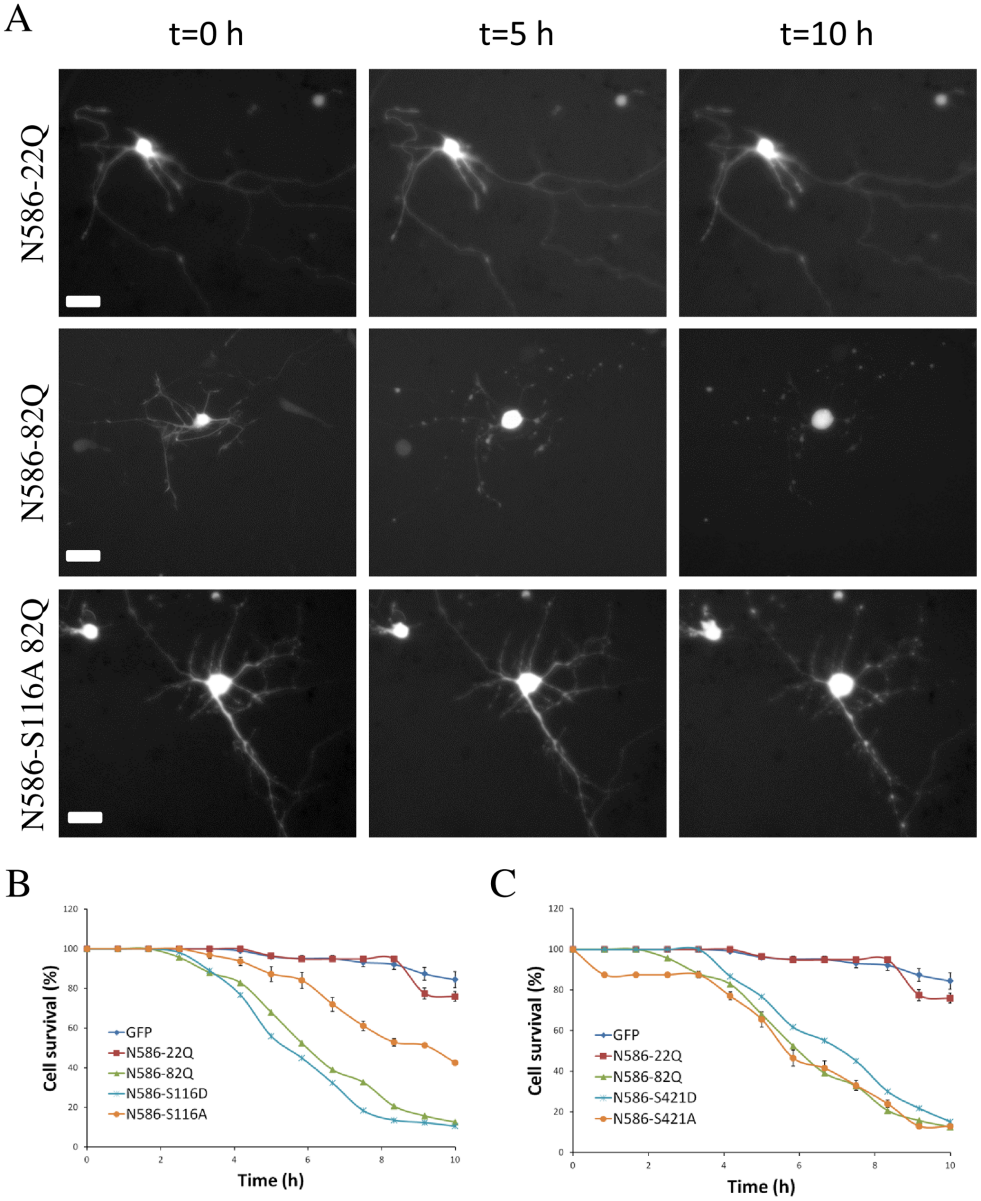
Time lapse imaging of toxicity of N586 constructs. Primary cortical neurons were co-transfected at DIV5. Beginning 24 hours after transfection, GFP positive neurons were imaged every 10 mn for 10 hours. (A) Representative images of cells transfected with N586-22Q (top row), N586-82Q (middle row), or N586-82Q S116A (bottom row) at t = 0 (left column), t = 5 h (center column), and t = 10 h (right column). (B) Quantification of cell survival. For each time point cells were given the value of 100 if alive and 0 if dead. Results are expressed as mean ± sem. n = 200 cells analyzed in 5 independent experiments.

In order to confirm the effect of the alterations of phosphorylation sites in expanded Htt on its toxicity and to demonstrate directly that these alterations affected Htt-mediated neuronal cell death in culture, we used a time lapse video microscopy assay. This method is comparable to a time lapse analysis method used in the Finkbeiner laboratory [68], [69], but is set up to be useable with standard microscopy facilities without custom software or robotics. The assay makes it possible to follow cells over time by video microscopy, in this case beginning at hour 38 and ending at hour 48 post-transfection, which is the same time point at which cell toxicity is measured using the nuclear condensation assay. At the beginning of the experiment, cells are still showing healthy morphology and deteriorate over time. The time frame of degeneration is consistent with the nuclear condensation assay which shows significant death only after 36 hours [32]. As shown in Figure 4A, this assay demonstrated robust neuronal cell death in neurons transfected with Htt-N586-82Q compared to neurons transfected with normal Htt or GFP alone. We observed a significant reduction in toxicity of Htt-N586-82Q S116A, closely paralleling the results in the initial screen using the nuclear condensation cell death assay (Figure 4B). As in the nuclear condensation assay, the phosphomimetic alteration S116D did not cause a dramatically change in Htt toxicity though there is a suggestion that there may be an increase in toxicity. In the time lapse assay the alteration at position 421 did not have a dramatic effect, but had a trend toward decreasing toxicity.

## Discussion

In this study we have used mass spectrometry to identify potential phosphorylation sites within the N-terminal region of Htt. We have identified novel potential sites, and have confirmed previously known S421 phosphorylation site. Using a nuclear condensation cell toxicity assay in primary neurons, we set up a screen to evaluate the effects of alterations of phoshorylation sites in polyQ-expanded Htt on its toxicity. Alanine replacement at one site, serine 116, caused a significant decrease of Htt-mediated cell toxicity. We confirmed this effect using a cell death assay based on time lapse video microscopy to directly track cell death.

Strengths of this study include the identification of several novel candidate phosphorylation sites, focusing on the N-terminus of Htt near the polyQ repeat, a region of the protein likely to be relevant for HD pathogenesis. We were able to determine a functional effect of an alteration of at least one of these sites.

There were several limitations of the study. The discovery experiments were all carried out *in vitro*, and relied on over-expression in non-neuronal cell lines. We did not completely cover the N-terminal 511 amino acids of Htt, though we did achieve 73.14% coverage of the region (excluding the previously studied N-terminal 17 amino acids, the polyQ repeat and the proline repeats, which do not contain any residues which could be phosphorylated). A number of the phosphorylation sites we found did not have high ion scores, however our goal was to be inclusive rather than exclusive in pursuing candidate sites, and then to use functional assays to identify sites with potential physiologic relevance.

We have included the phosphorylation sites within the N-terminal 17 amino acid region in the functional studies because of the previous reports of protective effects of Htt phosphorylation within this region. Although, because of the unfavorable configuration of tryptic cleavage sites in this region, we did not identify Htt phosphorylation sites within its N-terminus. Similar to previous studies, we found that phospho-mimetic alteration at the S13/16 sites ameliorated toxicity.

We also studied the previously reported phosphorylation at S421 and confirmed the neuroprotection [50] with replacement by aspartate. Unlike one previous study, however, we did not find that substitution of S421 by alanine had no effect. By contrast we found suggestion of some protection. The reason for this discrepancy is not clear at the moment. Phosphorylation of Htt at S421 by Akt and SGK kinases has been reported to reduce Htt toxicity, and to restore axonal transport in neurons [47], [50]. Our results, demonstrating that alteration preventing phosphorylation of S421 reduce Htt neuronal toxicity seems to contradict this notion. This raises a possibility that S421D mutation may not mimic phosphorylation, and that both alterations prevent phosphorylation of S421, which turns out to reduce Htt toxicity, at least in our system. We wonder if the previous experiments with Akt and SGK kinases might have found neuroprotection due to other effects of the kinases besides direct effects on Htt itself. Certainly Akt is well known to be part of signaling pathways promoting global cellular survival.

Neuronal cell death is highly relevant to pathogenesis of HD, and cell loss in postmortem HD striatum best reflects premortem motor impairment and functional disability [67]. This assay has the disadvantage of being relatively low throughput, but feasible for the relatively small number of sites we had to study. The only alteration which significantly altered expanded Htt toxicity was S116A; however some of the other alterations, such as T107A and the cluster at 457/459/461/464 showed trends, and might be followed up in future studies.

The S116A alteration showed significant and reproducible protection in the nuclear condensation assay. This was clearly supported by the data from the time-lapse microscopy data, which showed substantial decrease of Htt-mediated cell death. It should be kept in mind that cell culture experiments with over-expressed protein involve an *in vitro* short-duration system, and will need *in vivo* confirmation. In our experiments, we saw protection with the serine to alanine alteration, but no change with the serine to aspartate alteration. This could indicate that the stoichiometry of phosphorylation at this site is relatively high or possibly that the aspartate substitution does not well mimic the effects of phospho-serine at this site.

In this study we have attempted to find phosphorylation sites with functional relevance that could be involved in disease pathogenesis. We found a striking effect of alteration of the S116 site on mutant Htt cellular toxicity. This raises the possibility that phosphorylation of S116 could be involved in HD pathogenesis. This would be reminiscent of other neurodegenerative diseases in which phosphorylation is known to modulate cellular toxicity of the relevant disease protein. For instance, in the case of SCA1, alteration of serine 776 in a transgenic mouse model substantially ameliorated the phenotype [70]. Phosphorylation of tau is involved in Alzheimer's disease and other taupathies, and is likely to have an important role in promoting AD pathogenesis [71]. Phosphorylation of α-synuclein at S129 has been implicated in PD pathogenesis [72].

Htt phosphorylation has been suggested to have potential therapeutic relevance. Small molecule modulation of phosphorylation within the first 17 amino acids alters Htt sub-cellular localization and Htt cleavage and toxicity [58]. Inhibition of calcineurin by FK506 also can have a protective role through increase of Htt phosphorylation at serine 421 [51], though targeting the m-TOR pathway with Everolimus did not have a similar effect [73]. Ganglioside GM1 induces phosphorylation of mutant Htt and ameliorates the abnormal phenotype in HD mice [63]. Thus, a better understanding of phosphorylation of Htt may provide therapeutic targets for treatment of HD.

The candidate phosphorylation sites identified here still need to be confirmed *in vivo*. However, if phosphorylation of Htt at serine 116 can be shown to be relevant *in vivo*, then we would propose that phosphorylation of S116 has an important modulatory role in HD pathogenesis. Because changing the serine to alanine is protective, inhibiting the kinase or kinases that phosphorylate this site should also be protective. Thus this phosphorylation event could be a candidate therapeutic target for HD.

## Supporting Information

Table S1 **Primer sequences for Htt-N586-82Q site directed mutagenesis.**
(DOCX)
