# Supplementary material for: Phosphorylation of Mutant Huntingtin at Serine 116 Modulates Neuronal Toxicity
Source: PLoS One. 2014 Feb 5;9(2):e88284. doi: 10.1371/journal.pone.0088284 (PMC3914950; doi:10.1371/journal.pone.0088284)
Supplement: Table S1 — Primer sequences for Htt-N586-82Q site directed mutagenesis. (DOCX) [file pone.0088284.s001.docx]

| T3A f | 5'CTAATTCACATGGCGGCCCTGGAAAAGC 3' |
| --- | --- |
| T3A r | 5'GCTTTTCCAGGGCCGCCATGTGAATTAG 3' |
| T3D f | 5'CCGCTAATTCACATGGCGGACCTGGAAAAGC 3' |
| T3D r | 5'GCTTTTCCAGGTCCGCCATGTGAATTAGCGG 3' |
| S13A f | 5'GAAGGCCTTCGAGGCCCTCAAGTCCTTCC 3' |
| S13A r | 5'GGAAGGACTTGAGGGCCTCGAAGGCCTTC 3' |
| S13D f | 5'GAAGGCCTTCGAGGACCTCAAGTCCTTCC 3' |
| S13D r | 5'GGAAGGACTTGAGGTCCTCGAAGGCCTTC 3' |
| S16A f | 5'CTTCGAGTCCCTCAAGGCCTTCCAGCAGC 3' |
| S16A r | 5'GCTGCTGGAAGGCCTTGAGGGACTCGAAG 3' |
| S16D f | 5'CTTCGAGTCCCTCAAGGACTTCCAGCAGC 3' |
| S16D r | 5'GCTGCTGGAAGTCCTTGAGGGACTCGAAG 3' |
| S13/16A f | 5'CTTCGAGGCCCTCAAGGCCTTCCAGCAG 3' |
| S13/16A r | 5'CTGCTGGAAGGCCTTGAGGGCCTCGAAG 3' |
| S13/16D f | 5'CGAGGACCTCAAGGACTTCCAGCAGCAG 3' |
| S13/16D r | 5'CTGCTGCTGGAAGTCCTTGAGGTCCTCG 3' |
| S95A f | 5'GCACCGACCAAAGAAAGAACTTGCAGCTACCAAGA 3' |
| S95A r | 5'TCTTGGTAGCTGCAAGTTCTTTCTTTGGTCGGTGC 3' |
| S95D f | 5'GCTGCACCGACCAAAGAAAGAACTTGATGCTACCAAGAAAGACCG 3' |
| S95D r | 5'CGGTCTTTCTTGGTAGCATCAAGTTCTTTCTTTGGTCGGTGCAGC 3' |
| T97A f | 5'CAAAGAAAGAACTTTCAGCTGCCAAGAAAGACCGTGTG 3' |
| T97A r | 5'CACACGGTCTTTCTTGGCAGCTGAAAGTTCTTTCTTTG 3' |
| T97D f | 5'GACCAAAGAAAGAACTTTCAGCTGACAAGAAAGACCGTGTGAATCATT 3' |
| T97D r | 5'AATGATTCACACGGTCTTTCTTGTCAGCTGAAAGTTCTTTCTTTGGTC 3' |
| S95/T97A f | 5'CAAAGAAAGAACTTGCAGCTGCCAAGAAAGACCGTGTG 3' |
| S95/T97A r | 5'CACACGGTCTTTCTTGGCAGCTGCAAGTTCTTTCTTTG 3' |
| S95/T97D f | 5'GACCAAAGAAAGAACTTGATGCTGACAAGAAAGACCGTGTGAATCATT 3' |
| S95/T97D r | 5'AATGATTCACACGGTCTTTCTTGTCAGCATCAAGTTCTTTCTTTGGTC 3' |
| T107A f | 5'GACCGTGTGAATCATTGTCTGGCAATATGTGAAAACATAGTGG 3' |
| T107A r | 5'CCACTATGTTTTCACATATTGCCAGACAATGATTCACACGGTC 3' |
| T107D f | 5'GAAAGACCGTGTGAATCATTGTCTGGATATATGTGAAAACATAGTGGCACAGT 3' |
| T107D r | 5'ACTGTGCCACTATGTTTTCACATATATCCAGACAATGATTCACACGGTCTTTC 3' |
| S116A f | 5'GAAAACATAGTGGCACAGGCTGTCAGAAATTCTCCAG 3' |
| S116A r | 5'CTGGAGAATTTCTGACAGCCTGTGCCACTATGTTTTC 3' |
| S116D f | 5'GAAAACATAGTGGCACAGGATGTCAGAAATTCTCCAG 3' |
| S116D r | 5'CTGGAGAATTTCTGACATCCTGTGCCACTATGTTTTC 3' |
| S116E f | 5'GTGAAAACATAGTGGCACAGGAAGTCAGAAATTCTCCAG 3' |
| S116E r | 5'CTGGAGAATTTCTGACTTCCTGTGCCACTATGTTTTCAC 3' |
| S120A f | 5'ACATAGTGGCACAGTCTGTCAGAAATGCTCCAGAATTTCAG 3' |
| S120A r | 5'CTGAAATTCTGGAGCATTTCTGACAGACTGTGCCACTATGT 3' |
| S120D f | 5'ACATAGTGGCACAGTCTGTCAGAAATGATCCAGAATTTCAG 3' |
| S120D r | 5'CTGAAATTCTGGATCATTTCTGACAGACTGTGCCACTATGT 3' |
| S116/120A f | 5'GAAAACATAGTGGCACAGGCTGTCAGAAATGCTCCAGAATTTCAG 3' |
| S116/120A r | 5'CTGAAATTCTGGAGCATTTCTGACAGCCTGTGCCACTATGTTTTC 3' |
| S116/120D f | 5'GAAAACATAGTGGCACAGGATGTCAGAAATGATCCAGAATTTCAG 3' |
| S116/120D r | 5'CTGAAATTCTGGATCATTTCTGACATCCTGTGCCACTATGTTTTC 3' |
| T271A f | 5'CACCATTCGGCGGGCAGCGGCTGGATCAG 3' |
| T271A r | 5'CTGATCCAGCCGCTGCCCGCCGAATGGTG 3' |
| T271D f | 5'CCCCCACCATTCGGCGGGACGCGGCTGGATCAGC 3' |
| T271D r | 5'GCTGATCCAGCCGCGTCCCGCCGAATGGTGGGGG 3' |
| S339A f | 5'CACAAGCCTGAAAGGCGCCTTCGGAGTGACAAGG 3' |
| S339A r | 5'CCTTGTCACTCCGAAGGCGCCTTTCAGGCTTGTG 3' |
| S339D f | 5'CAAGCCTGAAAGGCGACTTCGGAGTGACAAG 3' |
| S339D r | 5'CTTGTCACTCCGAAGTCGCCTTTCAGGCTTG 3' |
| S421A f | 5'GGCCGAAGCCGTAGTGGGGCAATTGTGGAACTTATAGCTGG 3' |
| S421A r | 5'CCAGCTATAAGTTCCACAATTGCCCCACTACGGCTTCGGCC 3' |
| S421D f | 5'GGCCGAAGCCGTAGTGGGGACATTGTGGAACTTATAGCTGG 3' |
| S421D r | 5'CCAGCTATAAGTTCCACAATGTCCCCACTACGGCTTCGGCC 3' |
| S434A f | 5'GGGGGTTCCTCATGCGCCCCTGTCCTTTCAAG 3' |
| S434A r | 5'CTTGAAAGGACAGGGGCGCATGAGGAACCCCC 3' |
| S434D f | 5'GAGGGGGTTCCTCATGCGACCCTGTCCTTTCAAGAA 3' |
| S434D r | 5'TTCTTGAAAGGACAGGGTCGCATGAGGAACCCCCTC 3' |
| S457A f | 5'GCCTTGGAGGATGACGCTGAATCGAGATCGG 3' |
| S457A r | 5'CCGATCTCGATTCAGCGTCATCCTCCAAGGC 3' |
| S457D f | 5'GCCTTGGAGGATGACGATGAATCGAGATCGG 3' |
| S457D r | 5'CCGATCTCGATTCATCGTCATCCTCCAAGGC 3' |
| S459A f | 5'GGAGGATGACTCTGAAGCGAGATCGGATGTCAGC 3' |
| S459A r | 5'GCTGACATCCGATCTCGCTTCAGAGTCATCCTCC 3' |
| S459D f | 5'CCTTGGAGGATGACTCTGAAGACAGATCGGATGTCAGCAGC 3' |
| S459D r | 5'GCTGCTGACATCCGATCTGTCTTCAGAGTCATCCTCCAAGG 3' |
| S461A f | 5'CTCTGAATCGAGAGCGGATGTCAGCAG 3' |
| S461A r | 5'CTGCTGACATCCGCTCTCGATTCAGAG 3' |
| S461D f | 5'GATGACTCTGAATCGAGAGACGATGTCAGCAGCTCTGC 3' |
| S461D r | 5'GCAGAGCTGCTGACATCGTCTCTCGATTCAGAGTCATC 3' |
| S464D f | 5'CGAGATCGGATGTCGACAGCTCTGCCTTAAC 3' |
| S464D r | 5'GTTAAGGCAGAGCTGTCGACATCCGATCTCG 3' |
| S465A f | 5'GATCGGATGTCAGCGCCTCTGCCTTAACAG 3' |
| S465A r | 5'CTGTTAAGGCAGAGGCGCTGACATCCGATC 3' |
| S465D f | 5'GATCGGATGTCAGCGACTCTGCCTTAACAGC 3' |
| S465D r | 5'GCTGTTAAGGCAGAGTCGCTGACATCCGATC 3' |
| S457/459A f | 5'GGAGGATGACGCTGAAGCGAGATCGGATGTC 3' |
| S457/459A r | 5'GACATCCGATCTCGCTTCAGCGTCATCCTCC 3' |
| S457/459D f | 5'AGAAGAAGCCTTGGAGGATGACGATGAAGACAGATCGGATGTCAGC 3' |
| S457/459D r | 5'GCTGACATCCGATCTGTCTTCATCGTCATCCTCCAAGGCTTCTTCT 3' |
| S457/459/ 461A f | 5'GATGACGCTGAAGCGAGAGCGGATGTCAGCA 3' |
| S457/459/ 461A r | 5'TGCTGACATCCGCTCTCGCTTCAGCGTCATC 3' |
| S457/459/ 461D f | 5'GGATGACGATGAAGACAGAGACGATGTCAGCAGCTCTGCCT 3' |
| S457/459/ 461D r | 5'AGGCAGAGCTGCTGACATCGTCTCTGTCTTCATCGTCATCC 3' |
| S457/459/ 461/464A f | 5'GCGAGAGCGGATGTCGCCAGCTCTGCCTTAAC 3' |
| S457/459/ 461/464A r | 5'GTTAAGGCAGAGCTGGCGACATCCGCTCTCGC 3' |
| S457/459/ 461/464D f | 5'GAAGACAGAGACGATGTCGACAGCTCTGCCTTAACAGC 3' |
| S457/459/ 461/464D r | 5'GCTGTTAAGGCAGAGCTGTCGACATCGTCTCTGTCTTC 3' |
| S457/459/ 461/464/ 465A f | 5'AGAGCGGATGTCGCCGCCTCTGCCTTAACAGC 3' |
| S457/459/ 461/464/ 465A r | 5'GCTGTTAAGGCAGAGGCGGCGACATCCGCTCT 3' |
| S457/459/ 461/464/ 465D f | 5'GACAGAGACGATGTCGACGACTCTGCCTTAACAGCCTC 3' |
| S457/459/ 461/464/ 465D r | 5'GAGGCTGTTAAGGCAGAGTCGTCGACATCGTCTCTGTC 3' |
| S461A f | 5'CTCTGAATCGAGAGCGGATGTCAGCAG 3' |
| S461A r | 5'CTGCTGACATCCGCTCTCGATTCAGAG 3' |
| S461D f | 5'GATGACTCTGAATCGAGAGACGATGTCAGCAGCTCTGC 3' |
| S461Dr | 5'GCAGAGCTGCTGACATCGTCTCTCGATTCAGAGTCATC 3' |
| S464A f | 5'GAATCGAGATCGGATGTCGCCAGCTCTGCCTTAACAG 3' |
| S464A r | 5'CTGTTAAGGCAGAGCTGGCGACATCCGATCTCGATTC 3' |
| S464D f | 5'CGAGATCGGATGTCGACAGCTCTGCCTTAAC 3' |
| S464D r | 5'GTTAAGGCAGAGCTGTCGACATCCGATCTCG 3' |
| S465A f | 5'GATCGGATGTCAGCGCCTCTGCCTTAACAG 3' |
| S465A r | 5'CTGTTAAGGCAGAGGCGCTGACATCCGATC 3' |
| S465D f | 5'GATCGGATGTCAGCGACTCTGCCTTAACAGC 3' |
| S465D r | 5'GCTGTTAAGGCAGAGTCGCTGACATCCGATC 3' |
| S487A f | 5'GCTTCTTCAGGGGTTGCCACTCCAGGGTC 3' |
| S487A r | 5'GACCCTGGAGTGGCAACCCCTGAAGAAGC 3' |
| S487D f | 5'CTGCTTCTTCAGGGGTTGACACTCCAGGGTCAGCA 3' |
| S487D r | 5'TGCTGACCCTGGAGTGTCAACCCCTGAAGAAGCAG 3' |
| T488A f | 5'CTTCAGGGGTTTCCGCTCCAGGGTCAGCA 3' |
| T488A r | 5'TGCTGACCCTGGAGCGGAAACCCCTGAAG 3' |
| T488D f | 5'CTTCTTCAGGGGTTTCCGATCCAGGGTCAGCAGGTC 3' |
| T488D r | 5'GACCTGCTGACCCTGGATCGGAAACCCCTGAAGAAG 3' |
| S487/T488A f | 5'GCTGCTTCTTCAGGGGTTGCCGCTCCAGG 3' |
| S487/T488A r | 5'CCTGGAGCGGCAACCCCTGAAGAAGCAGC 3' |
| S487/T488D f | 5'GCTGCTTCTTCAGGGGTTGACGATCCAGGGTCAGC 3' |
| S487/T488D r | 5'GCTGACCCTGGATCGTCAACCCCTGAAGAAGCAGC 3' |

Table S1: Primer sequences for Htt-N586-82Q site directed mutagenesis
